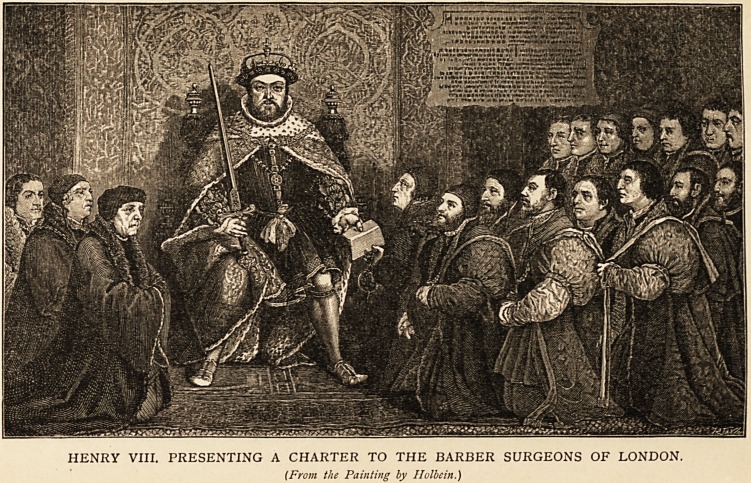# Shakspere and the Medical Sciences

**Published:** 1887-12

**Authors:** L. M. Griffiths


THE BRISTOL

### Medico-Chirurgical Journal.

DECEMBER, 1887.

### SHAKSPERE AND THE MEDICAL SCIENCES.

The Presidential Address at the opening, on October 12th, 1887, of the

14th Session of the Bristol Medico-Chirurgical Society,

BY

L. M. GRIFFITHS, M.R.C.S. Eng., L.R.C.P. Ed.

---

IT has almost become a stereotyped plan for the president of a society like ours to introduce into his address, by way of preface, a statement, more or less in detail, that for the fulfilment of the duties of the office he is much less capable than any of his predecessors. This is probably an outcome of the retiring modesty of the Profession in its individual capacity and of the desire to carry out the apostolic precept of esteeming others better than one's self. If it were absolutely true—and if it were a fact that the welfare of the constitution depended upon the nominal president—a society which had got to its fourteenth session with a steady deterioration constantly going on would indeed be in a sad state.

I shall try to avoid the mistake of offering this customary apology, because to doubt the wisdom of the choice of the members seems to me to cast a slur upon the intelligence of the electing body, and I think it is more becoming, when elected, to enter manfully into the duties of the office and do the best that in one lies.

But the choice of the subject of an address involved considerable difficulty. Rather than bore you by a quasi-philosophical dissertation; or try your patience with a sort of clinical lecture; or even attempt to instruct you by unverified semi-scientific hypotheses, I decided that I would seek to interest you by presenting, after independent investigation, a record of what Shakspere has to say about Medical Men and their modes of procedure. As of late years circumstances have brought me into a tolerably close familiarity with the Shaksperian text, I felt that I might, in a new way, say something about the matter which, if it did not profit, might, at least, not weary. The end of the sixteenth and the beginning of the seventeenth century was a period richer in intellectual force than any previous to our own age; and it seemed to me that it might possibly have a general interest if I fixed your attention upon the views of current medicine as expressed in the writings of the greatest observer of human nature in all its aspects. It would any way absolve me from the charge of slavishly following in the steps of any of my distinguished predecessors, and it might, in a brief space, show something of the development of one period in our medical history.

We are not justified in ignoring the history of any period of the development of our art. Our views three hundred years hence may appear crude and barbaric to a generation that may have reduced medicine to an exact science, and that may have invented a race of medical men able to apply the principles of their profession with a precision exceeding even that of the automatic machine of the present day. An automatic doctor is one of the possibilities of the future. But looking out from our temporary residence in Hades, we shall feel wronged if the share, small as it may be, that we have had in attaining this end is not taken into account.

This Society is engaged in the high and responsible duty of doing its best to alleviate present suffering, and also of helping forward the knowledge by which a posterity shall find the exercise of its art more easy and more scientific. This should make us careful not to advance theories on insufficient data, but only after a well-considered study of facts presented to our observation. Amidst much good scientific work of the present day there is too great a tendency to hasty generalisation. An earnest professor in an obscure continental Clinic, in a small series of cases of a certain disease, administers a new drug, in spite of which the patients get well. In an elaborate essay bristling with chemical formulæ the remedy is vaunted as a specific, but upon extended experience is fated to be relegated to that collection of broken reeds to which the practitioner has unfortunately trusted when he has temporarily forgotten that he has to treat a disease *plus* an individuality.

We need at present more records from men whose minds are fitted to test the balance of evidence, and who are unbiassed in favour of particular views. Our hospitals are not affording us the information we have a right to expect from them. It is much to be desired that com-

17 *

petent experts should be entrusted with the task of comparing and summarising hospital-results, which have been carefully prepared by trained observers. An attempt in this direction was made in the plan introduced some years ago, and known as "Collective Investigation." But there the great error was made of appealing individually to men too limited in their experience of particular diseases; and the fallacy of such a mode was mercilessly, but opportunely, exposed by an anonymous writer in our own Journal some years ago;* and, fortunately for the medical scientific reputation of the last quarter of the nineteenth century, but little is now heard of that ill-begotten scheme.

It was only by an accumulated observation of facts that such fixed articles as we have in our medical creed have got into their definite form. There is, therefore, a peculiar fitness in going, for the subject of an address, to a writer who has given us a truer picture of the time in which he lived than any one else has been able to present, and this by holding the mirror up to Nature and then noting in expressive language the facts which he observed.

It has been claimed for Shakspere that in some branches of medical science he showed a knowledge far in advance of members of the profession. On the other hand, it has been asserted that he knew no more than any old woman of the period could have told him. It was my purpose to collect the passages referring, directly or indirectly, to medical science or practice, and thus try to find where the truth lies between these conflicting statements; for a third hypothesis—that he may, for the medical allusions, have consulted some practitioner of the healing art—can be dismissed at once, as the references

* *Bristol Medico-Chirurgical Journal*, Vol. II., pp. 196–9. 1884.

are too numerous and too incidental for such a theory to be taken into consideration.*

But when I had partly put this intention into practice, I found that I had marked † so many passages for comment that, if I had carried out my original plan, I should have had to have written an address far exceeding the usual length of such discourses.

It seemed undesirable to make selections, and so I determined to limit the subject of this address to Shakspere's references to **The Practitioner**, trusting to the Editor to find me, in future numbers of the Journal, space for the comments on the other passages.

Shakspere brings before us six classes of persons engaged in what we now recognise as branches of our Profession. These are—**The Physician, The Surgeon, The Apothecary, The Tooth-drawer, The Midwife, The Nurse.** In addition to these, and closely connected with them in Shakspere's day, there is **The Gatherer of Simples.**

Dr. Bucknill "arrived at the fullest conviction that the great dramatist had, at least, been a diligent student of all medical knowledge existing in his time." ‡ And referring to *Much Ado about Nothing* (IV. i. 254),§ he says,

* I believe that such a practice was adopted by the late "Hugh Conway" in his widely-read *Called Back*, and that he obtained the information required from a member of our Society.

† It is almost needless to say that I could not have done this without the help of Mrs. Cowden-Clarke's *Concordance* and Schmidt's *Lexicon*, two indispensable works.

‡ *The Medical Knowledge of Shakespeare*, p. 290. London, 1860. This is a book which every doctor, and every Shakspere-student, ought to have. I have been greatly indebted to it. With the exception of Paré's book, it has been my only source for allusions in Shakspere's medical contemporaries.

§ All the line-references are to the "Globe" edition.

concerning the Friar's observation to Leonato about the appropriateness of using extreme methods for extreme diseases, "the passage is evidently copied from the sixth aphorism of Hippocrates," and he thinks Shakspere "derived it from some work on the original."*

It is of interest therefore to see how Shakspere refers to the names of those connected by fame with the healing art. Taking them in chronological order, we first find two of the most commonplace allusions to that very mythical personage, the God of Medicine. Cerimon, the physician in *Pericles,* when he hopes by his skill to prevent Thaisa having a relapse, says, as in duty bound—

"And Æsculapius guide us!"—III. ii. 111.

And the doctor in *The Merry Wives of Windsor* is saluted by the host in mock heroics as—

"My Æsculapius."—II. iii. 29.

Pythagoras, although not strictly a physician, deserves inclusion in the list because he influenced modes of life so greatly by introducing into his philosophic system such serious dietetic restrictions. Shakspere mentions him upon three occasions, each in connection with his doctrine of transmigration of souls—a theory for which Shakspere had no respect. Gratiano, moved by Shylock's vindictiveness, says to him:

"Thou almost mak'st me waver in my faith
To hold opinion with Pythagoras,
That souls of animals infuse themselves
Into the trunks of men: thy currish spirit
Governed a wolf, who, hanged for human slaughter,
Even from the gallows did his fell soul fleet,
And, whilst thou lay'st in thy unhallowed dam,
Infused itself in thee."

*The Merchant of Venice,* IV. i. 130–7.

* *Op cit.,* p. 116.

Rosalind, amused at the verses in her praise found on the trees in the forest, says:

> "I was never so be-rhymed since Pythagoras' time, that I was an Irish rat."—*As You Like it*, III. ii. 186–7.

And the Clown, in his assumed ecclesiastical examination of Malvolio, catechises him thus:

> CLOWN. "What is the opinion of Pythagoras concerning wild fowl?"
> MALVOLIO. "That the soul of our grandam might haply inhabit a bird."
> CLOWN. "What thinkest thou of his opinion?"
> MALVOLIO. "I think nobly of the soul, and no way approve his opinion." *Twelfth Night*, IV. ii. 54–60.

But it is not to his influence upon medical practice that his views are of much interest to doctors who, perhaps unconsciously, have had more to do with him as a geometrician, notably as the originator of the forty-seventh proposition of the first book of Euclid.

To Hippocrates, Shakspere has only one allusion by name, and that in a perverted form by Sir Hugh Evans, who depreciates the worth of the "renowned French physician," whom he describes as ill read in the works of the Father of Medicine. All laymen have a desire to know something of medical matters; and it is more than likely that in Shakspere this desire was so intensified that he would avail himself of all opportunities of looking into medical writings for a wider range of view, with the inevitable result of making a jumble in his own mind. How far his medical and surgical allusions are imbued with the spirit of Hippocrates, I must leave to the opinion of my hearers who are deeply read in both authors; but it may be not too much to say, that if Hippocrates had been an Elizabethan dramatist, the style of his writing would have been Shaksperian; and had Shakspere been a

Greek physician, his characteristics would have been those of Hippocrates, as a close observer and recorder of signs and a frugal prescriber of drugs. But in the surgery of the present day, Hippocrates would be quite at home; for more than two thousand years ago he was incising the pleura for the relief of empyema, and treating phthisical cavities by direct surgical operation, and practising cerebral surgery with much frequency.* On account of the veneration paid to the dead body by the Greeks, his opportunities for dissection were small. As therefore he was not hampered by a very elaborate knowledge of anatomy, it is perhaps fortunate for his reputation that there are on record no statistical tables of his results.

From him Shakspere might have learned much, for medical art in the sixteenth century had deteriorated sadly from its high position as expounded by Hippocrates.

Shakspere has two references to Aristotle, but each time as an ethical teacher, taking no cognisance of the fact that he, the son of a physician, had some medical tastes, and knew much of comparative anatomy.

Nearly six hundred years after Hippocrates came Galenus, whose influence was strong in Shakspere's day. In 1559—five years before Shakspere was born—Dr. John Geynes, the year before his admission as a Fellow of the Royal College of Physicians of London, "was cited before the College for impugning the infallibility of Galen. On his acknowledgment of error, and humble recantation signed with his own hand, he was received into the College." † So of course Shakspere has something to say about Galen. He is mentioned twice in *The Merry*

* *The Genuine Works of Hippocrates*, 2 vols. Sydenham Society. 1849.

† *The Roll of the Royal College of Physicians of London.* 2nd edition. Vol. I., p. 62. 1878.

*Wives of Windsor* (II. iii. 29, and III. i. 67); once as a companion to Æsculapius in the host's appeal to the doctor already mentioned, and the other time associated with Hippocrates as an author in whom, according to Evans, Master Doctor Caius showed such lamentable ignorance. In *All's Well that Ends Well* (II. iii. 12), there is an allusion to his leadership of a school of medicine, where the wonder is expressed that the king could have been cured after being "relinquished by the artists" who practised after the manner of Galen. He was also the authority whence Sir John Falstaff had derived his muddled knowledge of apoplexy (2 *Henry IV.*, I. ii. 133). In *Coriolanus,* Shakspere uses the name of Galen as an opportunity for a scoff at medical practice, in which he was fond of indulging, as the opinion that he always paid respect to the profession is not borne out by his references. Menenius, whose spirits are raised by the news that Coriolanus is coming home after his victory at Corioli, drags in, without the least appropriateness, this sneer at the profession :

> "A letter for me! it gives me an estate of seven years' health; in which time I will make a lip at the physician: the most sovereign prescription in Galen is but empiricutic, and, to this preservative, of no better report than a horse-drench."
> II. i. 127-30.

Dr. Bucknill's comment on this passage is typical of the mode of thought which sees an appreciative testimony in every allusion that Shakspere makes to doctors. He says: "Menenius describes the health preserving effect of the pleasure it affords him, in terms which convey the poet's high appreciation of Galen, the great medical authority of his own day."* The fact that Coriolanus, if he ever lived at all, had been dead nearly six hundred

* *Op. cit.*, p. 207.

years when Galen was born did not trouble Shakspere. Galen at the present day would have been an excellent family doctor. He had a high opinion of Hippocrates, whose surgical prowess he does not seem to have emulated, although he was much given to surgery which did not involve operation. Galen is the last great name in Medicine for many centuries, and very little advance seems to have been made in the art till well on into the nineteenth century.

Paracelsus, who is coupled with Galen as one whose followers were not equal to curing the fistula of the king in *All's Well that Ends Well,* must be looked upon as a bit of a quack, although by his knowledge of chemistry he added considerably to the resources of pharmacy. It was through him that calomel and opium were generally used internally; but although he had a nostrum which would secure him from the fate of death, it did not keep him alive more than eight-and-forty years. Shakspere most appropriately names Paracelsus as the representative of a school opposed to that of Galen, whose works Paracelsus had publicly burned. But it was not only those specifically named as of the schools of Galen and Paracelsus who had given up the king's case, but he had also been "relinquished of all the learned and authentic fellows that gave him out incurable." (II. iii. 14, 16.) These, who would be disciples of Galen, had been previously referred to by the king himself as "our most learned doctors," and he goes on to say:

> "The congregated college have concluded
> That labouring art can never ransom nature
> From her inaidable estate." II. i. 120-2.

When Shakspere wrote the words "congregated college," no recent institution was in his mind. The College of Physicians had been established in 1518, and the nearest act of incorporation of a medical body had been that in 1540, which united the Barbers' Company and the Guild of Surgeons as the Company of Barbers and Surgeons.

The king in *All's Well that Ends Well* is a French king, but that would not have prevented Shakspere from putting in English touches. But the congregated college to which he alludes probably has no definite meaning, although in 1603 "the College of Physicians in the University of Paris, being lawfully congregated," not only judged Turquet de Mayern unworthy to practise because he had publicly identified himself with the tenets of the chemical school of Paracelsus, but forbade all who were of their "Society" to hold consultation with him.

The mention of this Frenchman brings one conveniently to Dr. Caius of *The Merry Wives of Windsor*, because it has been sought to show that this "renowned French physician," as Master Page in the play calls him, was intended by Shakspere to represent Turquet de Mayern, who was known to English people as Sir Theodore de Mayerne. But Mayern did not settle in England till 1610, although he was here in 1606. He was appointed first physician to James I., and took a high place in professional life. He was a man of moderate views, and was able to see good both in the views of the old-fashioned practitioners and the chemical reformers. He was about the last man in the world that Shakspere would have burlesqued as the doctor of *The Merry Wives;* and as the first sketch of the play (in which Caius appears) was printed in 1602, it may be concluded that there is no connection between the two personages.

An attempt has been made to show that Shakspere meant to portray the well-known Dr. Caius whose name is connected with a "munificent foundation at Cambridge."

But Dr. Caius was a man held in high honour, whom there was no occasion or need to satirise. He was an Englishman born, and when the play was written he had been dead thirty years.

This fancy may also be completely dismissed. The truth probably is, that Shakspere wanted to poke fun once again at a Frenchman, and took this name as that of a doctor well remembered, but about the details of whose history he knew little and cared less.

There is one point which seems to connect the doctor in *The Merry Wives* with Caius of Cambridge. The doctor and the Welsh parson are by no means friendly in their inter-communications. "Dr. Caius in the statutes of the college founded by him specially excludes persons who are Welshmen from holding any of his Fellowships." * But this is probably a mere coincidence.

The only actual living doctor that Shakspere introduces among his *dramatis personæ* is Dr. Butts, physician to Henry VIII. He appears, not in his medical capacity, but as a sympathiser with Cranmer disrespectfully treated by his judges. The king, when he hears from Butts the details of this treatment, becomes more strongly than before the friend and advocate of Cranmer. The portrait of Dr. Butts, whom Henry VIII. knighted, is preserved in a picture † by Holbein, which is now in the Hall of the Barbers' Company. Butts is the foremost figure of the three on the right of the king. Henry VIII. was generous with other people's property to an extent perhaps unequalled by any other being, and

* Hunter's *Illustrations of Shakespeare*, Vol. I., p. 210. 1845.

† For a representation of this, taken from South's *Memorials of the Craft of Surgery in England*, I am indebted to the courtesy of Messrs. Cassell and Company. South's book is full of interest for doctors. A notice of it appeared in the *Bristol Medico-Chirurgical Journal*, Dec., 1886.

HENRY VIII. PRESENTING A CHARTER TO THE BARBER SURGEONS OF LONDON.

*(From the Painting by Holbein.)*

on Butts he bestowed rich gifts of abbey-lands. Medical knights of to-day have to be content with the bare honour.

In addition to these actual personages, Shakspere has many creations of medical men; and in one of these instances he is an example that might at the present day be occasionally followed with much propriety. In *Pericles*, he who tells us (III. ii. 31-2) that "I ever have studied physic" is a lord of Ephesus. A medical peer is to us in England such a creature of the imagination that it is difficult to draw him in definite outline, and much more so to fill him in with colours. But it was not so shortly after Shakspere's day. A noble contemporary of his, who outlived him many years, has the honour of having his name associated with Medicine. Henry, Lord Marquis of Dorchester, Earl of Kingston-upon-Hull, and Viscount Newark, after a long illness which familiarised him with doctors and physic, at the age of forty-three brought his great talents to bear upon the study of Medicine, and he became a Member, and then a Fellow, of the Royal College of Physicians. He nearly met his death by inadvertence. Dr. Goodall, in a MS. which is in the College of Physicians' Library, says: "In the morning, as soon as he was out of bed, he did often use to take a cordial electuary of his own prescribing; and at this time calling hastily for it, his stomach not being very well, the woman that kept it, amongst many other things of this and the like kind, by her over-diligence and haste mistook the gallipot, and instead thereof brought a pot of the extractum cardiacum, an excellent medicine taken in a due proportion; but he took so large a dose of it that his physicians judged he had taken near 100 grains of opium, which is one ingredient that medicine is compounded of. Within less than a quarter of an hour he grew heavy and dozed, and so into a dead sleep. This mistake was not discovered for three hours; when presently his coach was sent from Highgate, where he was then at his house, for Sir John Micklethwaite and Dr. Browne, with an account of this accident, who presently repaired to him, and found him in all appearance never to be recovered; the medicine was dispersed into the habit of his body, and they thought he would depart in this sleep; but using their utmost endeavours, by forcing down something to make him vomit, and a clyster into his body, he did evacuate plentifully downwards, and after twenty-four hours came somewhat to himself again, and in three or four days' time to good understanding."* The case is not recorded with Hippocratic exactness; but if it represents the facts, we have an instance of a medical peer showing a tolerance of a drug which certainly has never been shown by a doctor of less exalted rank. The day seems far distant when we may have the opportunity of trying such a dose on another medical member of the House of Lords, although our life-saving profession ought to have been one of the first from which additions should have been made to that august assembly. If the tenure of the Presidential Chair of this Society were to be rewarded with a well-endowed peerage, it would not be too great a recompense for the labour which it has cost some of us to prepare our introductory addresses.

In addition to this medical marquis, who was a great benefactor of his college, it is stated by Dr. Bucknill, who quotes from Ward's *Diary*, that "Edmund, Earl of Derby, who died in Queen Elizabeth's days, was famous for chirurgerie, bone-setting and hospitalitie."† I have

* *The Roll of the Royal College of Physicians.* 2nd edition. Vol I. pp. 289–90. 1878. † *Op. cit.*, p. 276.

found no other mention of his professional habits, which were probably of an amateur character. In our own times we have a member of a continental Royal family practising a specialty with much success.*

The practitioners introduced by Shakspere are physicians who, as a class, are still, in popular estimation, higher in repute than surgeons. The reason of this is not difficult to discover. The clergy in early days monopolised all professions, and were the depositaries of everything that was good, and, in later monastic times, of much that was bad. After practising surgery for a long period, the religious sentiment became offended by the shedding of blood, and a papal edict † went forth that no operations were to be performed which involved such a result. The medical ecclesiastics, whilst rendering obedience to their spiritual authority, were wise in their generation; no longer able to perform the surgical operations themselves, they determined to retain some hold over the procedure. Barbers, who were largely employed for tonsorial purposes, seemed to furnish a class intellectually enough lower than the clergy to be kept in submission, and yet possessing that steadiness of hand and familiarity with cutting instruments which would render them ready pupils in such operations as may be required. These operations were carried out in the presence of those who were restricted to the medical part of the profession, and so well was their relation maintained, that notwithstanding the aggressive efforts of the successors of the monastic barber, and the forefathers of the modern surgeon, the College of Physicians, as late as 1632, "procured an order of council with a clause to the effect that no chirurgeon 'doe either dis-

* *Good Words.* July, 1887.

† By Innocent III., in 1215.

member Trephan the head, open the chest or Belly, cut for the stone, or doe any great opperation with his hand uppon the body of any person to which they are usually tyed to call their Wardens or Assistants, but in the presence of a learned physitian one or more of the College or of his Maj<sup>ties</sup> physitians;' "* and it was not till 1635 that this order was cancelled by Charles I. Such was the abject condition of the operating surgeon in Shakspere's days, and therefore it is no wonder that all his medical personages are physicians. The steps by which the developed monastic shaver was able to attain a position by which he could throw off this yoke, I will touch upon when I come to Shakspere's allusions to Surgeons.

In the year 1607, when Shakspere was forty-three years of age, his daughter Susanna married Dr. John Hall, who was in practice at Stratford. Collier thinks that when Shakspere came back to Stratford and settled in 1608 in New Place—the house which he had bought in 1597, and in which he died in 1616—that Dr. Hall and his wife lived there with him. In his will Shakspere left New Place to Mistress Hall, and there is positive evidence they were living there the year after Shakspere died. Dr. Hall was in good and large practice, as we know from the names of those whom he attended, of whom he speaks in the book, " Select Observations on English Bodies or Cures Empirical and Historical Performed on very eminent Persons in Desperate Disorders. First written in Latine by Mr. John Hall, Physician, of Stratford, where he was very famous, as also in the counties adjacent. Now put into English for common benefit by James Cook, Practitioner in Physick and Chirurgery. 1657." This was twenty-two years after Dr. Hall's death.

* South's *Memorials of the Craft of Surgery in England*, p. 215. 1886.

Confirmation of the belief that Dr. Hall took a high position is also found in the fact that his daughter—his only child—married, as her second husband, John Bernard, who was afterwards knighted by Charles II. in 1661; and in 1669, in the person of Lady Bernard, the lineal descendants of the poet came to an end.

No man is a hero to his own valet, and probably no doctor is a hero to his own father-in-law, especially if they live together. If this was true in reference to Shakspere and Dr. Hall, it would go far to explain some of the slighting and needless allusions to medical practice that so frequently appear in the plays.

I will now run through the list of Shakspere's physicians, taking the plays in their approximately chronological sequence, in order to see if, with the maturity of his powers, he saw any reason to regard them in varying lights.

In *The Comedy of Errors*, Pinch, described as a schoolmaster, takes upon himself the functions of an alienist physician, and is appealed to by Adriana to restore her husband to the senses which she supposed he had lost, as by that time in the play (Act IV.) he had got considerably mixed up with his twin brother, whom he accurately resembled. Pinch professes to diagnose the complaint by the state of the pulse and the pale and deadly look, and then, by means of his holy prayers, proceeds to remove the devil by whom he considers the man to be possessed. Finding this does not remove a non-existent disease, he orders restraint in a dark room—the routine treatment of lunacy—about which I shall have something more to say under the head of Mental Disease. Pinch is described as a schoolmaster,* one of a class who, being of superior

* The offices of schoolmaster and exorciser of spirits were often combined in one person. See references in Ben Jonson's *The Staple of News*, I. ii. and III. ii.

education, were credited with the power of dealing with spirits. In *Hamlet*, Marcellus requests Horatio to converse with the ghost of Hamlet's father, saying, "Thou art a scholar; speak to it, Horatio." From these passages it is seen that it was not the invariable rule to address spirits in Latin. Pinch is an unadulterated specimen of a humbug, who endeavoured to make capital by assuming powers which he did not possess. He is graphically described as

> "a hungry lean-faced villain,
> A mere anatomy, a mountebank,
> A threadbare juggler and a fortune-teller,
> A needy, hollow-eyed, sharp-looking wretch,
> A living-dead man." V. i. 237–41.

In him Shakspere exposes the irregular practitioner rather than the true physician.

In *Romeo and Juliet*, Friar Laurence does some impossible amateur doctoring in administering a drug to Juliet which, amongst other wonderful effects, can stop the pulse for two-and-forty hours. Shakspere, no doubt, fully believed this, which he took from the poem of *Romeus and Juliet*, translated from the Italian of Bandello by Arthur Brooke.* Friar Laurence, who thus unites two professions, recalls the early monks, who were ecclesiastics and doctors. His observations on the plants and flowers he gathers will be more appropriately considered under the head of Materia Medica.

*The Merry Wives of Windsor* is the earliest play in which there is a doctor among the characters. Here is Dr. Caius, to whom I have already made some reference. Dismissing all fanciful allusions to actual individuals, I shall look upon him simply as a portraiture of a practitioner with whom Shakspere came into contact, and in which he caricatures

* "No pulse shall goe, ne hart once beate within thy hollow brest,
But thou shalt lye as she that dyeth in a traunce."

the pretensions of the medical profession. It must be remembered that English practitioners were then very intolerant of foreigners who came here to practise their art, especially if they were bigger charlatans than themselves. In *The Return from Parnassus*, a University play in which Shakspere is mentioned by name, occurs the expression, "We 'll gull the world that hath in estimation forraine phisitians." Caius is introduced (I. iv. 45) sending for his "*boitier vert*" or his "green-a box." What this contained is doubtful. It may have had some instruments or appliances,* but more probably contained drugs,† which he could administer and charge for on the spot. He makes a brag of his surgical powers, and he threatens to remove the testicles of Sir Hugh Evans (I. iv. 118), whose interference in his love-matters he strongly resents. This is a piece of surgical braggadocio. His line is more correctly described by the language of the host, who, when the thought comes across him that his doctor may be killed in the duel, says, "Shall I lose my doctor? No; he gives me the potions, and the motions." (III. i. 104–5.) Probably in his vocation as landlord of the Garter Inn, he found a free and frequent purgation exceedingly beneficial.

Viewed in the light of clinical investigation of to-day, there are one or two references to Caius and his practices —connected with allusions in other plays—that are of great interest. Caius is called "bully stale" (II. iii. 30), "a Castalion-King-Urinal" (II. iii. 34), "Mounseur Mock water" (II. iii. 60); and about his head Evans twice

* Cf. *Troilus and Cressida*, V. i. 12.

† See l. 65. In the 1602 version of the play Caius sends for "de oyntment," and says:

"O I am almost forget
My simples in a boxe."

(III. i. 14, 91) threatens to knock his "urinals." In *The Two Gentlemen of Verona* (II. i. 39–43), Speed, telling his master of the evident signs of love which he shows, says "these follies are within you and shine through you like the water in an urinal, that not an eye that sees you but is a physician to comment on your malady." Falstaff, anxious about himself, sends a specimen of his urine to the doctor, and the messenger comes back telling him that the doctor said "the water itself was a good healthy water; but for the party that owed it, he might have more diseases than he knew for." (2 *Henry IV.*, I. ii. 3-6.) In *Twelfth Night* (III. iv. 114), Fabian and the others, desirous of knowing the condition of Malvolio and the prognosis, agree to "carry his water to the wise woman." Macbeth, with a sense of his country's impending danger, metaphorically says to his wife's medical attendant:

> "If thou couldst, doctor, cast
> The water of my land, find her disease,
> And purge it to a sound and pristine health,
> I would applaud thee to the very echo."
> V. iii. 50–3.

When we consider the importance which is now attached to examination of the urine, it appears strange that the attempt to thus diagnose disease, to which all these are references, should have brought its practitioners into great disrepute and infamy. But the fact is, that they attempted to be wiser than they actually were, and this is always a dangerous proceeding. They professed to name the complaint from a mere inspection of the secretion; and to such an extent was this practised, that the College of Physicians had more than once to interfere and frame statutes concerning it. If these "learned and authentic fellows" were to rise from their graves, it would probably take a long time to convince them that the multiplicity of tests applied to the urine now-a-days were being used with scientific precision. But even the results of modern investigations have been over-valued; to take an instance—the mere discovery of albumen has not the same importance attached to it that it had a few years ago. "Stale," in the phrase "bully stale," is another word for urine. Cæsar, lamenting over Antony's emasculated powers, recalls the time when, in the hardships of warfare which he had borne uncomplainingly, Antony had drunk "the stale of horses." (*Antony and Cleopatra*, I. iv. 62.) The urinal referred to in *The Merry Wives of Windsor* and *The Two Gentlemen of Verona* was the glass vessel in which the urine was reserved for inspection. It was learnedly known as the *matula*.

The intention to present Malvolio's urine to the judgment of "the wise woman" shows that this branch of professional work was also carried on by women in Shakspere's days, who, as a sex, had practised Medicine in earlier times. The modern return to this old custom, coming to us as a novelty, brought with it a shock from which we now seem to be recovering, and it was accompanied by a violence of language that was much to be deplored.

In *All's Well that Ends Well*, the reputation of Gerard de Narbon, a man famous in his profession as doctor, is mentioned. His remedies were such that they did not require a skilled person to apply them, for they were successfully used by his daughter, who had inherited his prescriptions; and his armamentarium was probably labelled for particular diseases, resembling some hospital preparations which were formerly known as "Mistura Tussis," "Mistura Febrifuga," and the like, and which could be administered by anyone who could turn the tap of the jar containing them. The details of the king's malady, and the conduct of his successful medical attendant, I shall refer to on another occasion. Shakspere took from Boccaccio the incident of the cure of the king by the daughter of a dead physician, when all the medical attendants he could obtain had failed. But his endorsement of the story cannot be considered a compliment to the profession.

The doctor in *Lear* is the first medical man in the plays for whom one does not entertain contempt. *Lear* must have been written about 1606; and as we have seen that Dr. Hall married Shakspere's daughter in 1607, it would seem that when the poet and the physician were brought more together, Shakspere put into a play, for the first time, a respectable practitioner, and not a burlesque representation of a doctor. This, of course, would not prevent him introducing some banter at doctors and their ways. Lear's medical attendant is only introduced to administer a sleeping-draught to the distraught patient. (IV. iv.) He has no marked individuality, and is peculiarly inoffensive; perhaps defers a little too much to his royal patient's daughter as to the time when he should awake the sick man; but Cordelia, as a sensible woman, gives him confidence by saying:

> "Be governed by your knowledge, and proceed
> I' the sway of your own will." IV. vii. 19, 20.

In *Macbeth* there are two doctors—an English one and a Scotch one. The English doctor appears only to announce that his king is coming forth to cure by touch "a crew of wretched souls" suffering from the disease here called "the evil." (IV. iii. 140-6.) Dr. Bucknill says the physicians were "either sufficiently ignorant or sufficiently polite, not to doubt, or not to appear to doubt, the possession by our kings of this miraculous therapeutic power." Shakspere gives the incident as he found it in Holinshed, from whom the historical record was taken. The Scotch doctor, like his modern antitype, is a shrewd practitioner. Coming to observe for himself the condition of Lady Macbeth, he refuses to take anything upon hearsay. He endeavours for the benefit of his patient to get the gentlewoman to tell him all she knows about the case, and then, when he is able to observe for himself, like a wise man, he makes a note at the time of what he hears. He is an honest man, and plainly says the case is not one for his treatment, as the symptoms all point to a distressed conscience; and this fact brings home to him his own shortcomings and sins, and pathetically he murmurs, "God, God, forgive us all." And then as a parting injunction he leaves an admirable piece of advice, that although the cause of the disease is beyond his reach, it may be aggravated by injudicious surroundings, and as long as her mind-torture leads her to walk in her sleep, she is exposed to physical dangers, and her attendants should "still keep eyes upon her." (V. i.) To the husband he reports in concise form the result of his enquiry, and reiterates his inability to deal with the case. The last we hear of him is a somewhat timid fear, excusable perhaps, that some foul play may be practised upon him, which even a big fee would not tempt him to risk by returning. (V. iii. 37–62.) Most likely Macbeth had not paid him anything. Altogether there is in this Scotch doctor much to be admired. His acts and demeanour would make the subject of an excellent address for students.

I have mentioned Cerimon in *Pericles* as the only one of Shakspere's doctors who can be classed among the nobility. His ways are, however, more amateurish than professional. His whole language is strained, and he is more a magician than a physician. He had

> "heard of an Egyptian,
> That had nine hours lien dead,
> Who was by good appliance recovered."
> III. ii. 84-6.

Dr. Bucknill gets over this difficulty by saying it is not to be taken literally, but that Shakspere meant "that the man had lain nine hours as dead." But a writer who could believe that there existed a drug of such marvellous power as that given to Juliet, could have no difficulty in bringing a man round who had been dead only nine hours. There is great doubt about the authorship of *Pericles*. It was not included in Shakspere's works till the third folio. I would fain believe that he had nothing to do with the creation of Cerimon.

Cornelius, described in *Cymbeline* as a physician, is upon his first appearance more of an apothecary. He is employed by the queen to bring poisons, with which she alleges she wishes to try experiments on the lower animals. He, good man, suspicious of her intent, gives her drugs which are only mild narcotics, and not what she imagines them to be. When he re-appears, at the end of the play, with the news of the queen's death, he seems to be more a friend of the family than a medical attendant.

Whilst the much-to-be-commended observation of the king in *All's Well that Ends Well* (II. i. 122-5)—

> "I say we must not
> So stain our judgment, or corrupt our hope,
> To prostitute our past-cure malady
> To empirics"—

may lead one to suppose that Shakspere had great respect for the authorised practitioner, it must be remembered that after all the king did that which he said he was not going to do, and that any way the importance of the passage is but comparative.

An examination of the following passages will show that Shakspere did not hold regular physicians in very high repute:—

Mistress Quickly, in *The Merry Wives of Windsor*, urging to Mistress Page the merits of Fenton as a suitor of her daughter Anne, said, referring to Caius:

> "Will you cast away your child on a fool, and a physician?"
> III. iv. 100–1.

Bertram gives expression to a similar statement:

> "A poor physician's daughter my wife!"
> *All's Well that Ends Well*, II. iii. 122.

Lafeu, giving to the Countess of Rousillon a reply to her enquiry for the king's health, says:

> "He hath abandoned his physicians, madam; under whose practices he hath persecuted time with hope, and finds no other advantage in the process but only the losing of hope by time."
> *All's Well that Ends Well*, I. i. 15–18.

Either Shakspere or Richard II. must have thought better of the physician's powers than of his morals, when the king, referring to old John of Gaunt, says:

> "Now put it, God, in the physician's mind
> To help him to his grave immediately."
> *Richard II.*, I. iv. 59, 60.

Timon says to the banditti:

> "Trust not the physician;
> His antidotes are poison, and he slays
> More than you rob."
> *Timon of Athens*, IV. iii. 434–6.

Posthumus, in *Cymbeline*, does not think much of medical efforts to cure the gout. Perhaps, if he were alive now, he would not find them much more successful. When in prison, he says:

> "Yet am I better
> Than one that's sick o' the gout; since he had rather
> Groan so in perpetuity than be cured
> By the sure physician, Death." V. iv. 4–7.

"Death will seize the doctor too" is such an obvious truism, that Cymbeline would certainly not have told Cornelius so (V. v. 29), unless with the intention of lowering the physician's self-importance.

And for the surgeon, Shakspere has the same uncomplimentary allusion. In *The Tempest,* when the passengers escaped from shipwreck are reviewing their position, Gonzalo, commenting on the inopportuneness of a remark of Sebastian's, says:

> "You rub the sore
> When you should bring the plaster."

Upon which Antonio adds, as a biting sneer:

> "And most chirurgeonly." II. i. 138–40.

Shakspere's references to surgeons are not to individual practitioners. One illustration is thereby afforded of the low estimation in which they were held in his day. Those whose merits were supposed to consist, as their name implies, in mere manual dexterity, were of course thought less of than those who had to apply mental processes to the determination and cure of disease. There was in Shakspere's time much need for the advocacy of Paré,* who in his "Works" eloquently, and with much acuteness, says:

"*Seeing there be three parts of Phyſick which at this time we profeſs;* Chirurgery, *which by the uſe of the hand*, Diet *which with the convenient manner of feeding and ordering the body*, *and* Pharmacy *that by Medicins attempt to expel Diſeaſes*,

* Paré died in 1590, just as Shakspere was beginning his literary life.

*and preſerve Health; The prime Phyſicians do not without reaſon contend which of theſe may be accounted the chief. Certainly* Herophilus *had* Pharmacy *in ſuch eſteem, that he thought Medicins were firſt mixed and adminiſtred to the Sick by* Apollo *(whom Antiquity thought a great Deity.) And* Pliny *had ſo great an opinion of* Diet, *that he exclaims, The true Remedies and Antidotes againſt Diſeaſes, are put into the Pot and eaten every day by the poor People. Verily all learned men confeſs, that the manner of curing which which is performed by Diet, is much more facil and proſperous than that which is done by Medicins; as thoſe things which ſought with much labour and coſt are taken with much loathing, and taken are ſcarce retained, but retained they oft work with much labour and pain: Which things long ago moved* Aſclepiades *to exclude the uſe of Medicins as hurtful to the ſtomach. Yet if we will believe* Celsus, *neither of theſe parts merit the preheminence, but both of them give place to* Chirurgery. *For ſeeing that Fortune is very powerful in Diſeaſes, and the ſame Meats and Medicins are often good and often vain, truly it is hard to ſay, whether the health is recovered by the benefit of Diet and Pharmacy, or by the ſtrength of the body. Moreover in thoſe caſes in which we moſt prevail with Medicins, although the profit be moſt manifeſt, yet it is evident that health is often ſought in vain even by theſe things, and often recovered without them. As it may be perceived by ſome troubled with ſore Eyes, and others with Quartan Fevers, who having been long troubled by Phyſicians, are healed without them. But the effect of Chirurgery as it is very neceſſary, ſo it is the moſt evident amongſt all the parts of Phyſick. For who without Chirurgery can hope to cure Broken or Luxated parts, who Wounds and Ulcers, who the Falling of the Matrix, the Stone in the Bladder, a Member infeſted with a Gangrene or Sphacele? Beſides, this part alſo is the moſt antient; for* Podalirius *and* Machaon *following their General* Agamemnon *to the* Trojan *Wars, yielded no ſmall comfort to their Fellow-Souldiers. Whom notwithſtanding* Homer *affirms not to have given any help in the Peſtilence, nor in divers*

*other Diſeaſes, but onely were accuſtomed to heal Wounds by Inſtruments and Medicins. And if the difficulty of learning it argue the excellency of the Art, who can doubt but Chirurgery muſt be the moſt excellent, ſeeing that none ought to be accounted a Chirurgeon, or which can perform his duty without the knowledge of* Diet *and* Pharmacy? *But both the other can perform their parts without Chirurgery, if we may believe* Galen. *But if we conſider the matter more nearly according to truth, we ſhall underſtand thoſe three parts have a certain common bond, and are very near of kindred, ſo that the one implores the aid of the other; neither can the Phyſician do anything praiſe-worthy without the conſpiracy and joint conſent of theſe three; therefore in ancient times there was but one Performer and Uſer of all the three Parts. But the multitude of men daily increaſing, and on the contrary, Mans life decreaſing, ſo that it did not ſeem able to ſuffice for to learn and excerciſe all the three, the Workmen divided themſelves. Wherefore that which happens to any man either by lot, or counſel, that let him follow, maintain and onely uſe, as mindful how ſhort his life is, and how long the Art.*"*

The barbers, whom the clergy had employed to perform operations, naturally, as time went on, were unwilling to continue their position as mere handicraftsmen, and were jealous of the success of those "surgeons who were not shavers." They succeeded, upon petition to the Court of Aldermen of the City of London, in getting their rights recognised by authority. Thus there were two authorised sets of men practising surgery—those licensed by the Guild of Surgeons, and those belonging to the Guild of Barbers. The Barber-surgeons went on improving their position, till, in 1540, in the reign of Henry VIII., an Act of Parliament was passed forming the Barbers' Company and the Guild of Surgeons into

* This extract is taken from the preface to the 1634 English edition The whole of the preface should be read.

the Company of Barbers and Surgeons. The union continued till 1745, more than two centuries. The difficulties of this incongruous alliance are well chronicled by South.*

In 1745 the separate Surgeons' Company was formed, and this lasted till 1796. In 1800 the Royal College of Surgeons of England was established by Royal Charter. Surgeons have not yet, in lay opinion, quite recovered from the relative inferiority engendered by attention being too greatly directed to their manipulative dexterity, and their long association with the barber.†

But the salvation of the surgeons, if we are to believe one authority, is to come from an unexpected quarter, and, as in the case of the re-uniting of the Colleges of Physicians and Surgeons, history is to repeat itself. At a meeting of the Hairdressers' Guild, held at St. James's Hall, London, the lecturer said:

> "Our services are indispensable, and the world cannot do without us. It is now more than 140 years since we separated ourselves from the surgeons, but there may come an age in which we shall be re-united." ‡

Irrespective of the definite acts the surgeon has done, or is to do, there are some general references to surgeons, but these are mostly of a commonplace or metaphorical character.

Duncan's injunction to get "surgeons" for the bleeding sergeant in *Macbeth* does not imply that there was need of assistance or consultation, but was probably only a part of that regal and noble magnificence which gives orders on a large scale, and which led Capulet to issue an order for twenty cunning cooks. In Lear's case, the desire to

* *Memorials of the Craft of Surgery in England.*

† A Boston man, who had evidently suffered much at the hands of his hairdresser, says that if surgeons are no longer barbers, many barbers are still surgeons. ‡ *Western Daily Press*, Oct. 2, 1886.

have "surgeons" was probably more real. Perhaps the most natural of all these indefinite allusions is where Mercutio, fatally stabbed by Tybalt, having called for a surgeon, goes on to say, in answer to Romeo's wish that the hurt cannot be much:

> "No, 'tis not so deep as a well, nor so wide as a church-door; but 'tis enough, 'twill serve: ask for me to-morrow, and you shall find me a grave man. I am peppered, I warrant, for this world. A plague o' both your houses! 'Zounds, a dog, a rat, a mouse, a cat, to scratch a man to death! a braggart, a rogue, a villain, that fights by the book of arithmetic! Why the devil came you between us? I was hurt under your arm. . . .
>
> Help me into some house, Benvolio,
> Or I shall faint. A plague o' both your houses!
> They have made worms' meat of me: I have it,
> And soundly too: your houses!"
>
> *Romeo and Juliet*, III. i. 99–113.

To another occasion I must leave the development of the Apothecary, with Shakspere's mention of him, and also all the references to the subsidiary branches of our profession.

Although it will have been gathered from what I have said that the extravagant claims put forward for Shakspere's insight into medical matters cannot be allowed, yet, as medical men, we should learn this much from him: that we shall be helping forward our art if we copy his faithfulness in noting the facts that come under observation; endeavouring, even if we can only do so in a lamentably imperfect way, to imitate his conciseness of language, and, by a careful study of his works, to breathe in something of the spirit of that mode of expression which his incomparable language has marked as the standard by which we should judge our own puny efforts, and to the height of which we should be always endeavouring to raise ourselves. Medicine of Shakspere's period must not be judged by the knowledge of to-day. If our art has not advanced since then, both in theory and practice, the sooner we leave our patients to the unalloyed benefits of the *vis medicatrix naturæ* the better for them. But those who seek to credit Shakspere with any special medical knowledge seem to me to do so to the disparagement of our profession,* which is not so poor a one that it can be expounded by him who has merely powers of observation and felicity of expression. There are fundamental principles (for instance, of anatomy and physiology) without an acquaintance with which the practitioner is walking a path beset with pitfalls, into which he is in constant danger of being entrapped. A knowledge of our profession cannot be obtained from books only: there must be clinical experience, with the voice of the living teacher to guide the learner through the intricacies of the elemental training. And of these necessities we know that Shakspere had none.

But, profiting by the example of his manner, let us go on accumulating facts, and be in no hurry to formulate theories. We have to possess our own souls—and other people's bodies—in patience. It is given to very few of us to do great things in our profession. It must be left to geniuses to systematise the observations of those who can be only humble labourers, and most of us will have to be content with being classed amongst these, who, however, are necessary in the economy of the world's progress.

Maybe there is not in the human body an undiscovered foramen or canal by which our name can be immortalised; and when painstaking efforts differentiate a new set of symptoms, or vanity leads to the modification, in a trifling

* This I hope to show in detail in another paper.

manner, of the existing satisfactory steps of some operation or mode of treatment, I hope we shall protest against the fashion of labelling such by the unscientific attachment of a personal name which to succeeding generations will connote nothing. By those who are to have an enduring influence, self must be kept in the background, and the attainment of scientific truth be made a constant study. Animated by these principles, we shall add both to the dignity and usefulness of our calling, and shall leave the world, professionally at all events, better than we found it.

My task to-night has been a humble one. It has not come within the scope of my capacity to fire your imagination with fanciful theories of healthy or morbid processes; or to record with a fervid enthusiasm the personal achievements of myself or others; or to dilate with a glowing imagery on the stupendous possibilities of the future; or to stir you to sudden mutiny because this or that fossilised corporation is said to inflict upon you wrongs of which you were not conscious till you were told about them. But if I have succeeded either in intensifying your regard for the history of a period fraught with the highest importance to our intellectual life or in directing your attention to one aspect of a great writer's work full of instruction and example for medical men; or even if I have incidentally renewed your interest in the (now lost) art of dramatic writing, in which it seems Shakspere is ever to stand without a rival, my ambition will have been more than satisfied.

## Figures and Tables

**Figure f1:**